# Substrate Specifity Profiling of the *Aspergillus fumigatus* Proteolytic Secretome Reveals Consensus Motifs with Predominance of Ile/Leu and Phe/Tyr

**DOI:** 10.1371/journal.pone.0021001

**Published:** 2011-06-17

**Authors:** Douglas S. Watson, Xizhi Feng, David S. Askew, Kalyani Jambunathan, Krishna Kodukula, Amit K. Galande

**Affiliations:** 1 Center for Advanced Drug Research, Biosciences Division, SRI International, Harrisonburg, Virginia, United States of America; 2 Department of Pathology & Laboratory Medicine, University of Cincinnati College of Medicine, Cincinnati, Ohio, United States of America; Duke University Medical Center, United States of America

## Abstract

**Background:**

The filamentous fungus *Aspergillus fumigatus* (*AF*) can cause devastating infections in immunocompromised individuals. Early diagnosis improves patient outcomes but remains challenging because of the limitations of current methods. To augment the clinician's toolkit for rapid diagnosis of *AF* infections, we are investigating *AF* secreted proteases as novel diagnostic targets. The *AF* genome encodes up to 100 secreted proteases, but fewer than 15 of these enzymes have been characterized thus far. Given the large number of proteases in the genome, studies focused on individual enzymes may overlook potential diagnostic biomarkers.

**Methodology and Principal Findings:**

As an alternative, we employed a combinatorial library of internally quenched fluorogenic probes (IQFPs) to profile the global proteolytic secretome of an *AF* clinical isolate *in vitro*. Comparative protease activity profiling revealed 212 substrate sequences that were cleaved by *AF* secreted proteases but not by normal human serum. A central finding was that isoleucine, leucine, phenylalanine, and tyrosine predominated at each of the three variable positions of the library (44.1%, 59.1%, and 57.0%, respectively) among substrate sequences cleaved by *AF* secreted proteases. In contrast, fewer than 10% of the residues at each position of cleaved sequences were cationic or anionic. Consensus substrate motifs were cleaved by thermostable serine proteases that retained activity up to 50°C. Precise proteolytic cleavage sites were reliably determined by a simple, rapid mass spectrometry-based method, revealing predominantly non-prime side specificity. A comparison of the secreted protease activities of three *AF* clinical isolates revealed consistent protease substrate specificity fingerprints. However, secreted proteases of *A. flavus*, *A. nidulans*, and *A. terreus* strains exhibited striking differences in their proteolytic signatures.

**Conclusions:**

This report provides proof-of-principle for the use of protease substrate specificity profiling to define the proteolytic secretome of *Aspergillus fumigatus*. Expansion of this technique to protease secretion during infection could lead to development of novel approaches to fungal diagnosis.

## Introduction

Filamentous fungi of the genus *Aspergillus*, most commonly *Aspergillus fumigatus* (*AF*), can cause devastating infections in immunocompromised individuals [1], [2], [3]. Mortality due to *AF* infection has doubled since 1980 and is strikingly high in chemotherapy-induced neutropenics (60%) and allogeneic bone marrow transplant recipients (90%) [4], [5]. Early diagnosis of *AF* infection enables timely therapy and is thus critical to a successful outcome [6], [7]. Unfortunately, many of the clinical signs of *AF* infection are nonspecific, and reliable early diagnosis remains challenging because of limitations in the sensitivity or specificity of existing tools, including mycological growth, radiology, detection of cell wall components by ELISA, and PCR amplification of fungal DNA [8], [9].

To augment the clinician's toolkit for rapid diagnosis of *AF* infections, we are investigating *AF* secreted proteases as novel diagnostic biomarkers. In nature, *AF* contributes to the recycling of carbon and nitrogen by degrading organic debris with numerous secreted hydrolases, including proteases [10]. Like many other environmental saprophytes, *AF* is highly specialized for this task, with more than 1% of its genome dedicated to the synthesis of secreted proteases alone [11], [12], [13], [14]. Metabolic studies have suggested that *AF* relies extensively on its ability to degrade protein to acquire essential nutrients during infection [15], [16], and these findings are consistent with the detection of *AF* proteases in the host environment [17], [18]. These enzymes degrade the extracellular matrix and disrupt the barrier function of the lung, allowing entry of the organism into the vasculature and hematogenous dissemination to other tissues [19], [20]. In addition, transcriptional profiling of *AF* isolated from infected mouse lungs revealed at least 11 protease-encoding mRNAs with greater abundance in vivo than in vitro, providing strong evidence that *AF* increases its repertoire of protease gene expression in the lung environment [21].

According to the MEROPS database, the *AF* genome encodes 136 proteases [22], predominantly serine/threonine proteases (39%) and metalloproteases (43%). Moreover, estimates of the number of secreted proteases range from 47 to 100 depending on the computational method used [10], [14], [19], [22], but fewer than 15 individual secreted proteases have been functionally characterized [23], [24], [25], [26], [27], [28], [29]. Given the large number of *AF* secreted proteases, the traditional reductionist approach of cloning and expressing individual enzymes has left most AF secreted proteases unexplored as diagnostic targets. In addition, the protease repertoire of AF is known to change in vivo [21], so the proteases that are most abundant in vitro may not be the most abundant or the most enzymatically active in vivo. Thus, a robust method for profiling the proteolytic signature of AF in vitro and in vivo addresses a deficit in the current literature and provides an alternative platform for discovery of AF secreted proteases as diagnostic biomarkers on the basis of enzymatic activity.

Activity-based protease substrate profiling provides a snapshot of the overall protease activity of a particular sample under a given set of conditions. Profiling techniques include active site-directed covalent probes [30], [31], which allow affinity purification of tagged enzymes; synthetic libraries of internally quenched fluorogenic probes (IQFPs), which fluoresce upon enzymatic cleavage [32], [33]; and phage display libraries, which are tethered to a solid surface and elute upon proteolysis [34]. These techniques are classically employed to define the substrate specificities of homogenous preparations of individual proteases [35], [36] or to purify individual components from mixtures [37], but they are not typically used to profile the proteolytic activities of complex samples.

**Figure 1:**
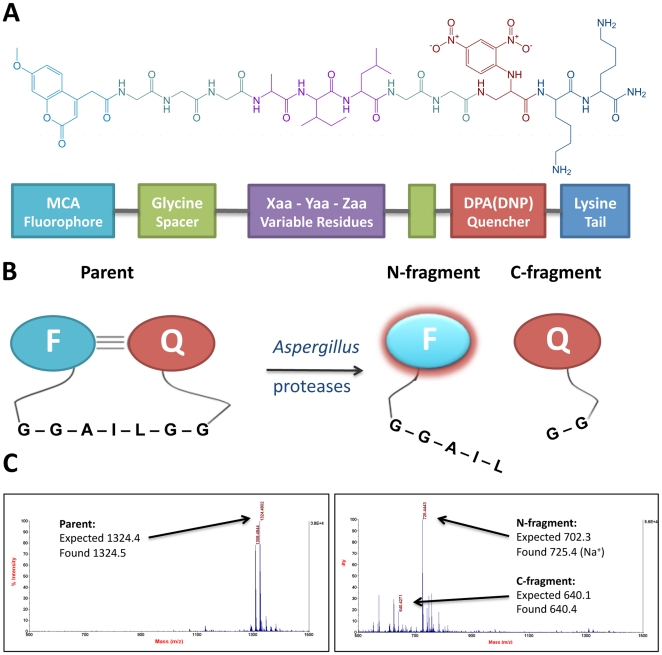
Strategy for protease activity profiling of *AF* secreted proteases. *A*. Structure and description of a typical IQFP probe from the library (variable sequence shown: Ala-Ile-Leu). *B*. Graphical depiction of IQFP cleavage by *AF* culture supernatant. N- and C-terminal substrate fragments were readily detected by MALDI-TOF mass spectrometry following ZipTip® C_18_ sample preparation. *C*. As an example, spectra of the sequence Ala-Ile-Leu before and after proteolytic cleavage are shown, with calculated fragments identified. A summary of all MS data is presented in Table 3.

We have recently reported a robust method for profiling protease substrate specificities of complex biological mixtures using a concise combinatorial IQFP library [38]. In this library, 15 amino acids that are commonly found in protease substrates are represented at each of three variable positions, flanked by appropriate spacer sequences and fluorophore or quencher moieties (Figure 1). The resulting 3375 IQFP sequences are combined into pools of up to 8 similar sequences and the resulting 512 peptide pools are arrayed in six 96 well microplates. Library “hits” are then deconvoluted by synthesizing the constituent IQFPs of a particular well to obtain fine substrate specificity information. In our previous study, we used this library to define the quantitative proteolytic fingerprints of two body fluids, bronchoalveolar lavage and serum, that have clinical relevance for diagnosis of *AF* infection.

In this study, we profiled the dominant substrate specificities of an *AF* clinical isolate in vitro. We also determined the functional characteristics of the *AF* proteases that cleave these substrates. Through comparative profiling of human serum, we showed that the proteolytic signatures of these *AF* secreted proteases and human serum are largely distinct. In addition, we observed little intraspecies variability in protease substrate specificities but found that multiple *Aspergillus* species could be distinguished through characteristic differences in their proteolytic signatures. These experiments represent an important first step toward the long-term goal of developing a diagnostic assay that detects *AF* infection through identification of the specific proteolytic signature of *AF* secreted proteases.

## Results

### Protease activity profiling of *AF* secreted proteases

Secreted proteases are novel targets for diagnosis of *AF* infection. Although serine endoproteases are predicted to be abundant among *AF* secreted proteases, only two have been characterized to date (Alp1 and SedA), only one of which is secreted at neutral pH (Alp1) [25], [27]. We reasoned that screening a combinatorial IQFP library against culture supernatant of an *AF* clinical isolate under classical serine protease buffer conditions would reveal an overall profile of the substrate specificities of *AF* secreted serine proteases. An initial comparison of buffer conditions of major protease classes (serin, metallo-, aspartic, and cysteine) using a generic fluorescent substrate (fluorescein-labeled casein) indicated that pH 8.0 gave the highest activity (data not shown), further supporting the rationale for screening under these conditions. Peptides in the IQFP library contain three variable residues, flanked on either side by a series of glycines, followed by either a fluorophore or a quencher (Figure 1). Each variable residue consists of an equimolar mixture of two closely related amino acids (for a more detailed description, see Materials and Methods). Thus, each well of the library contains an equimolar mixture of up to eight individual IQFPs. For example, the well containing the motif Ser/Thr–Ile/Leu–Asn/Gln contains the following eight sequences: Ser-Ile-Asn, Ser-Ile-Gln, Ser-Leu-Asn, Ser-Leu-Gln, Thr-Ile-Asn, Thr-Ile-Gln, Thr-Leu-Asn, and Thr-Ile-Gln.

**Figure 2:**
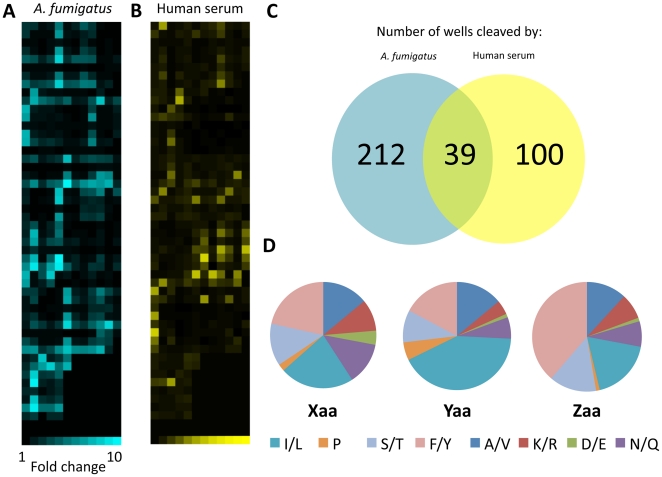
Substrate specificity of *AF* secreted proteases and human serum. *A*. Cleavage of an IQFP library by *AF* H237 culture supernatant. Each square corresponds to a single well of a 96 well microplate. The brightness of a given square corresponds to its fluorescence intensity, quantified as fluorescence fold change, which in turn indicates the extent of cleavage of the IQFP probes in that well. Of the 512 wells in the library, 93 exhibited greater than 4-fold fluorescence enhancement upon incubation with *AF* culture supernatant. *B*. Cleavage of the same IQFP library by complement preserved normal human serum. C. Number of IQFP sequences distinctly cleaved by only *AF* culture supernatant, only human serum, or both. *D*. Graphical depiction of amino acid preferences at each variable position of the library sequences cleaved by *AF* secreted proteases. Isoleucine, leucine, phenylalanine, and tyrosine were predominant at each position. Amino acid preference data are summarized in Table 1.

Comparative protease activity profiling of *AF* H237 culture supernatant and complement preserved normal human serum revealed striking differences in their proteolytic signatures (Figure 2A–B). Of the 512 wells in the library, 212 exhibited greater than 2-fold fluorescence enhancement upon exposure to *AF* culture supernatant but were not detectably cleaved by human serum (Figure 2C). In addition, 93 library wells exhibited greater than 4-fold fluorescence enhancement upon exposure to *AF* culture supernatant. 92 of these 93 substrate motifs contained isoleucine, leucine, phenylalanine, or tyrosine at one or more variable positions; 46.2% of the sequences contained one of these residues at one position, 45.2% at two positions, and 7.5% at all three positions (Figure 2D). These four residues accounted for 44.1% of *Xaa*, 59.1% of *Yaa*, and 57.0% of *Zaa* (Table 1). Positively charged residues (Lys, Arg) accounted for less than 10% of the residues at each variable position, hydroxyl-containing residues (Ser, Thr) less than 15%, negatively charged residues (Asp, Glu) less than 5%, and proline less than 5%. Of the 93 wells exhibiting 4-fold fluorescence enhancement or greater, several motifs (two constant positions and one variable position) appeared five or more times (Table 2). Four of the five motifs contained Ile/Leu at the *Yaa* position. The *Xaa* and *Zaa* positions were more varied but hydrophobic residues still predominated at the fixed positions of all five motifs except one, which contained Ser/Thr at the *Xaa* position.

**Table 1:** Amino acid preferences of IQFP sequences cleaved by *AF* secreted proteases.

	*Abundance at each variable position (%)*			*Number of instances per hit (% of all hits)*		
*Residues*	*Xaa*	*Yaa*	*Zaa*	*Once*	*Twice*	*Three times*
I/L	22.6	41.9	18.3	54.8	11.8	1.1
F/Y	21.5	17.2	38.7	47.3	14.0	1.1
A/V	14.0	14.0	11.8	29.0	5.4	0.0
S/T	12.9	9.7	14.0	30.1	3.2	0.0
N/Q	12.9	6.4	7.5	22.6	2.1	0.0
K/R	9.7	4.3	7.5	19.3	1.1	0.0
D/E	4.3	1.1	1.1	6.4	0.0	0.0
P	2.1	5.4	1.1	8.6	0.0	0.0

Left panel - for each possible residue, abundance at each of the three variable positions is reported. Right panel - the number of occurrences per IQFP sequence for each residue (once, twice, or three times) is shown as a percentage of all hits.

**Table 2:** IQFP motifs cleaved by *AF* secreted proteases.

*Xaa*	*Yaa*	*Zaa*	*Frequency*	*Fold Change*	*Selected Sequence*
Variable	F/Y	F/Y	5	7.25	I/L – F/Y – F/Y
A/V	I/L	Variable	5	7.11	A/V – I/L – I/L
S/T	I/L	Variable	6	5.01	S/T – I/L – F/Y
Variable	I/L	F/Y	5	4.91	N/Q – I/L – F/Y
F/Y	I/L	Variable	6	4.31	None selected

Motifs appearing at least five times among the 93 hits are shown. From each of the four motifs exhibiting the highest mean fold change fluorescence enhancement, a representative well not cleaved by human serum was selected for deconvolution and further analysis.

**Figure 3:**
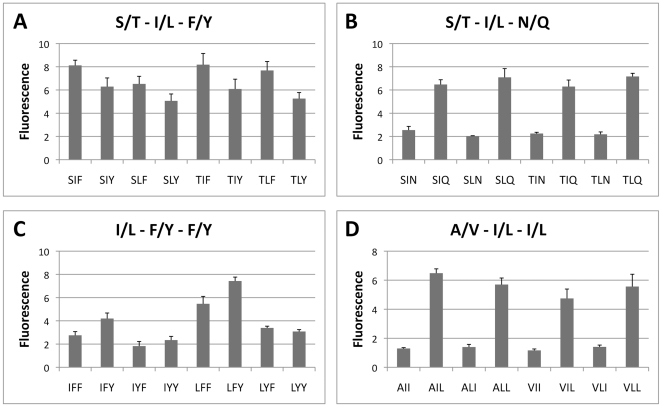
Deconvolution of *AF* protease substrate consensus motifs. For each IQFP motif with five or more wells demonstrating proteolytic cleavage by *AF* secreted proteases (listed in Table 2), a representative well was selected for deconvolution and further study. Individual IQFP sequences were synthesized and fine substrate specificity was determined. Although cleavage of the Ser/Thr–Ile/Leu–Phe/Tyr motif was promiscuous (*A*), others exhibited clear amino acid preferences, particularly at the *Zaa* position (*B, D*). Data represent fluorescence fold change after 2 h incubation.

**Figure 4:**
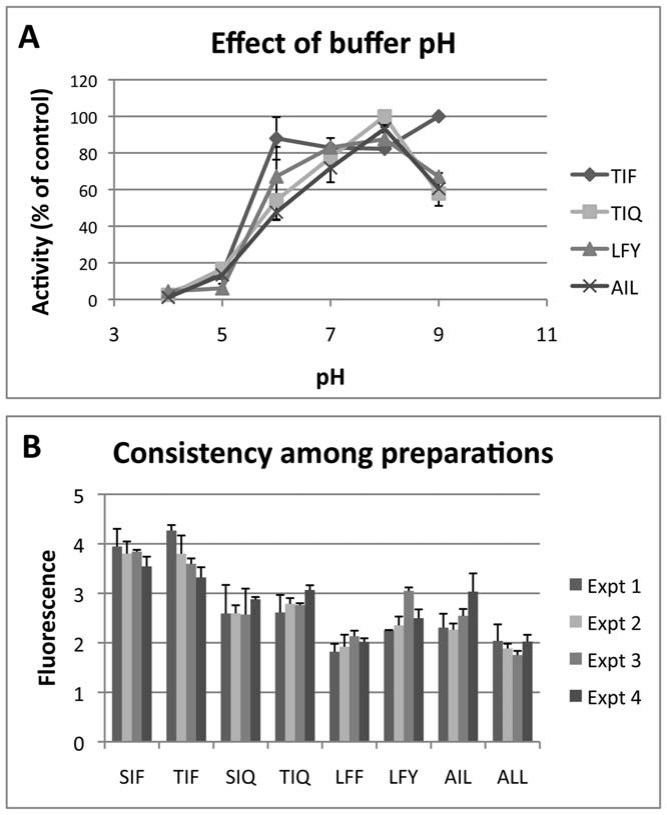
Properties of *AF* secreted proteases. *A*. Effect of buffer pH on activity of *AF* secreted proteases. Cleavage of each of the IQFP consensus motifs was optimal at approximately pH 8. Cleavage of three probes (Thr-Ile-Gln, Lue-Phe-Tyr, Ala-Ile-Leu) showed a narrow activity peak at pH 8 and a loss of activity at pH 9, whereas cleavage of Thr-Ile-Phe was near maximal in a broad range of assay buffer pH values. *B*. Cleavage of 8 IQFPs by 4 independent preparations of *AF* H237 culture supernatant. Proteolytic activities did not differ significantly among preparations.

From each of the first four IQFP motifs shown in Table 2, a representative well that was not cleaved by human serum was selected for further study. The IQFPs selected were: Ile/Leu–Phe/Tyr–Phe/Tyr, Ala/Val–Ile/Leu–Ile/Leu, Ser/Thr–Ile/Leu–Phe/Tyr, and Ser/Thr–Ile/Leu–Asn/Gln. The eight constituent sequences of each well were individually synthesized and confirmed by mass spectrometry. Fine amino acid substrate specificity was determined by quantifying endpoint fluorescence fold change following incubation of *AF* culture supernatant with each individual substrate (Figure 3). Three of the four motifs demonstrated a clear preference at one or more variable positions. The Ser/Thr–Ile/Leu–Asn/Gln motif required glutamine at the *Zaa* position, whereas the Ala/Val–Ile/Leu–Ile/Leu motif required leucine at the *Zaa* position. The Ile/Leu–Phe/Tyr–Phe/Tyr motif required phenylalanine at the *Yaa* position and also preferred tyrosine at the *Zaa* position. In contrast, the 8 constituent peptides of the Ser/Thr – Ile/Leu – Phe/Tyr motif were cleaved to a similar extent without dramatic preferences at any of the variable positions. From each motif, the two most efficiently cleaved IQFP sequences were selected for further characterization: Ser-Ile-Phe, Thr-Ile-Phe, Ser-Ile-Gln, Thr-Ile-Gln, Leu-Phe-Tyr, Leu-Phe-Phe, Ala-Ile-Leu, and Ala-Leu-Leu. Cleavage of each of these IQFPs was optimal at pH 8.0, although some exhibited broader pH maxima (Ser-Ile-Phe, Thr-Ile-Phe, Leu-Phe-Phe, Leu-Phe-Tyr) than others (Ser-Ile-Gln, Thr-Ile-Gln, Ala-Ile-Leu, Ala-Leu-Leu) (Figure 4A). Cleavage of these sequences was consistent across multiple independent culture preparations (Figure 4B).

### Properties of *AF* secreted proteases

MALDI-MS is a valuable tool for identification of biomarkers in complex samples [39], [40]. In this study, precise proteolytic cleavage sites of individual IQFPs were reliably determined by a simple, rapid mass spectrometry-based method without the need for liquid chromatography fractionation (Figure 1). At the conclusion of appropriate proteolytic cleavage assays, samples were purified using ZipTip® C_18_ pipette tips and directly eluted onto MALDI target plates. Assignment of cleavage sites based on predicted N- and C-terminal IQFP fragments revealed that three of the four motifs were cleaved at the *Zaa*-Gly bond, suggesting that the substrate specificity arises from non-prime side residues (Table 3). However, the Leu-Phe-Phe and Leu-Phe-Tyr sequences were cleaved at the *Yaa-Zaa* bond, indicating a contribution of both prime side and non-prime side residues to specificity.

**Table 3:** Assignment of IQFP cleavage sites by mass spectrometry.

		*Parent peptide (Da)*		*N-fragment (Da)*		*C-fragment (Da)*	
*Sequence*	*Cleavage site*	*Calc*	*Found*	*Calc*	*Found*	*Calc*	*Found*
SIF	SIF↓G	1374.4	1374.6	752.3	775.3 (Na^+^)	640.1	640.3
TIF	TIF↓G	1388.5	1388.6	766.3	789.3 (Na^+^)	640.1	640.3
SIQ	SIQ↓G	1355.4	1355.6	733.3	756.3 (Na^+^)	640.1	640.3
TIQ	TIQ↓G	1369.4	1369.6	747.3	770.3 (Na^+^)	640.1	640.3
LFF	LF↓FG	1434.5	1434.6	665.2	688.2 (Na^+^)	787.3	787.3
LFY	LF↓YG	1450.5	1450.5	665.2	688.2 (Na^+^)	803.3	803.3
AIL	AIL↓G	1324.4	1324.5	702.3	725.4 (Na^+^)	640.1	640.4
ALL	ALL↓G	1324.4	1324.5	702.3	725.2 (Na^+^)	640.1	640.4

Calculated and found masses of parent, N-fragment, and C-fragment peptides are shown. All N-terminal fragments were found with Na^+^ adducts. Two sequences per IQFP motif were analyzed.

**Figure 5:**
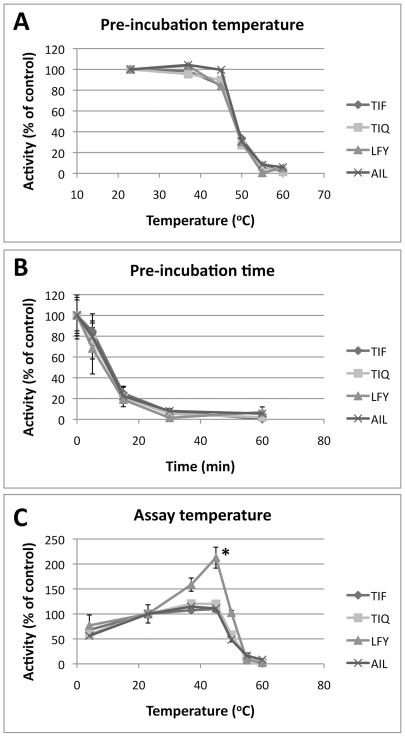
Thermostability and thermophilicity of *AF* secreted proteases. *AF* culture supernatants were heated before (*A,B*) or during (*C*) IQFP cleavage assays. *A*. *AF* culture supernatant was heated for 30 min at the indicated temperatures before addition of IQFPs. Proteolysis of each sequence sharply declined after heating at 50°C and was undetectable after heating at 55°C. *B*. *AF* culture supernatant was heated at 56°C for the indicated times before addition of IQFPs. Proteolysis of each sequence decreased markedly after heating for 15 min and was undetectable after heating for 30 min. *C*. *AF* culture supernatant and IQFPs were pre-heated for 15 min, combined, and incubated at the indicated temperature. Fluorescence was promptly measured after 1 hr. All IQFPs exhibited slightly increased cleavage at 45°C as compared to room temperature except LFY, which increased significantly. For all panels, data represent fluorescence fold change after 1 hr incubation. * p≤0.01 compared to room temperature.

Since enzymes derived from thermotolerant organisms are frequently thermostable, we explored the thermal properties of the secreted proteases responsible for these proteolytic activities with the long-term goal of using these characteristics to distinguish fungal proteases from their mammalian counterparts. When *AF* culture supernatant was heated for 30 min at variable temperature before starting the assays, cleavage of all four IQFP motifs retained full activity up to 45°C before exhibiting a sharp drop at 50°C and losing all detectable activity at 55°C (Figure 5A). Accordingly, incubation at 56°C for 15 min reduced activity by 80% (Figure 5B). To determine the effect of temperature on enzymatic activity, culture supernatant and IQFPs were combined and heated for 1 h before measurement of fluorescence. In this assay, three of the four IQFP motifs exhibited a slight, non-significant increase in activity up to 45°C and subsequent drop at 50°C, as compared to activity at room temperature (Figure 5C). Cleavage of Leu-Phe-Tyr, however, was significantly increased at 45°C and activity at 50°C was essentially equivalent to activity at room temperature. This difference in thermal properties, combined with a disparity in prime side substrate specificity, suggests that this proteolytic activity could arise from a distinct secreted protease as compared to the other IQFP motifs analyzed.

**Figure 6:**
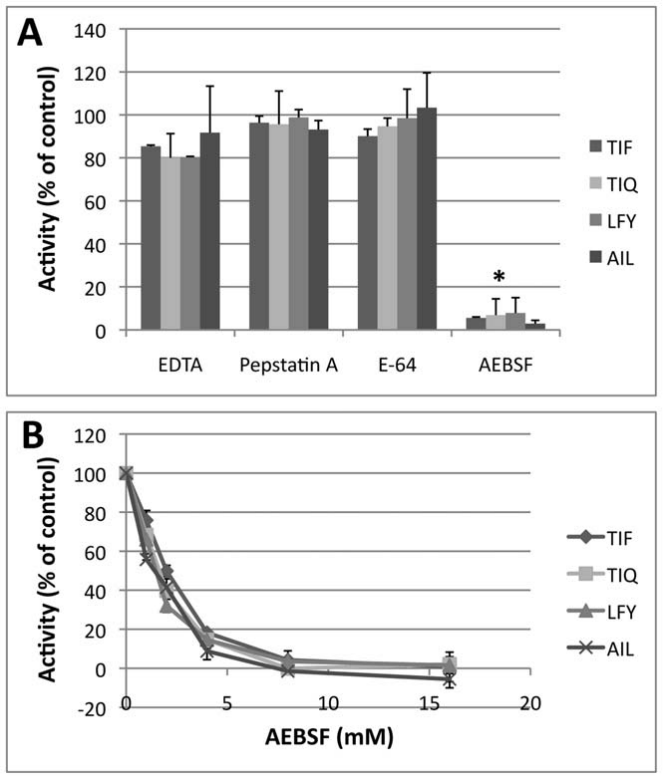
Inhibition of *AF* secreted protease activity by a serine protease inhibitor. *A*. AEBSF (8 mM) completely inhibited cleavage of all four IQFP motifs. No inhibition was observed with a metalloprotease inhibitor (EDTA; 20 mM), an aspartic protease inhibitor (Pepstatin A, 20 µM), or a cysteine protease inhibitor (E-64; 20 µM). *B*. The effect of AEBSF was dose-dependent. For each IQFP, data are normalized to a positive control sample lacking inhibitor. For both panels, data represent fluorescence fold change after 1 h incubation. * p≤0.05 for all IQFPs as compared to each of the three inhibitors and the positive control.

To test the hypothesis that our IQFP library screening conditions would identify serine protease activity, we determined the effects of class-specific protease inhibitors on cleavage of individual IQFPs by *AF* culture supernatant. A serine protease inhibitor, AEBSF, completely inhibited proteolytic activity of all four motifs in a dose-dependent manner (Figure 6). A metalloprotease inhibitor (EDTA), an aspartic protease inhibitor (Pepstatin A), and a cysteine protease inhibitor (E-64) had no effect when assayed at the high end of the range of typical inhibitor concentrations. Therefore, we conclude that the observed proteolytic activities arise from serine proteases.

### Proteolytic activity of *Aspergillus* species and strains

Distinguishing among *Aspergillus* species may be clinically useful because species can differ in their resistance to some antifungals [41]. Although the genomes of common *Aspergillus* species each encode more than 100 proteases [22], substantial differences in overall protease activity have been reported [10], [42], which could serve as a basis for distinguishing among species in a diagnostic assay. It is also important to determine the extent to which proteolytic activities vary among strains within a particular species. Thus, we compared the proteolytic profiles of culture supernatants derived from three strains each of *A. fumigatus*, *A. flavus*, and *A. nidulans*, along with one *A. terreus* strain. Proteolytic activities were normalized by dry weights of fungal cultures to account for slight differences in growth. *A. niger* was excluded from the comparison because it grew poorly under the conditions used for this study.

**Figure 7:**
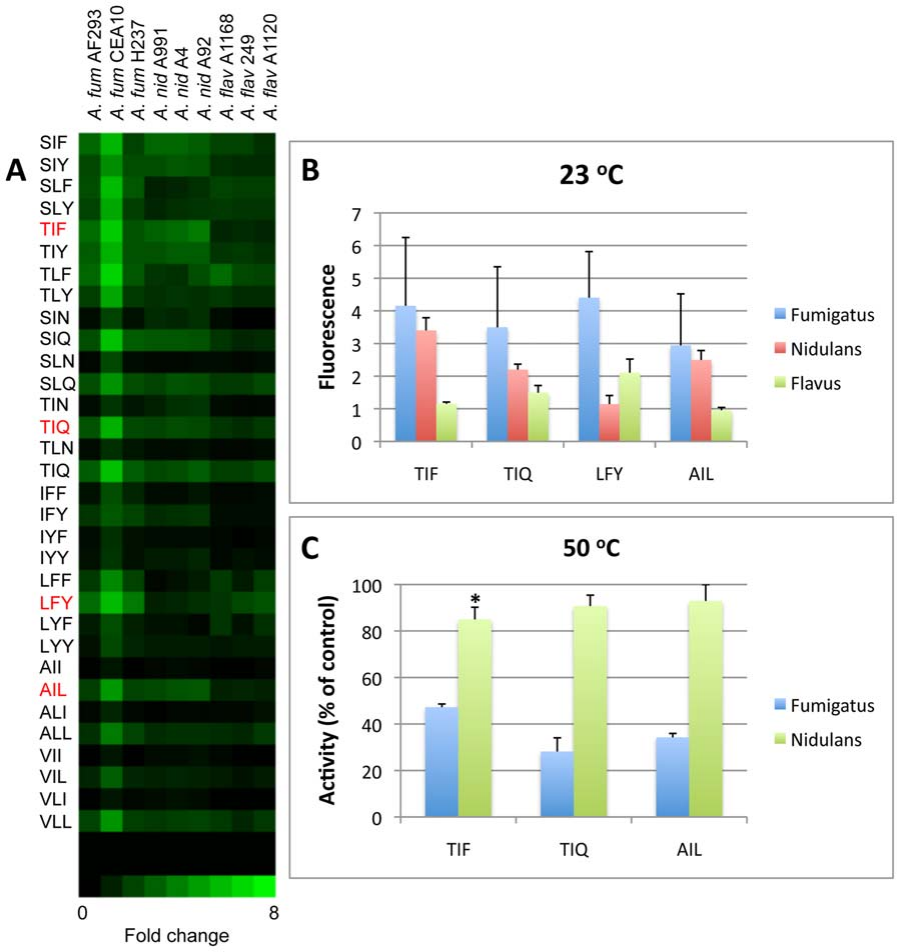
Comparative activity of secreted proteases of *Aspergillus* strains and species. *A*. Cleavage of individual IQFPs by *Aspergillus* culture supernatants. The brightness of a given square corresponds to the extent of cleavage of the IQFP probe in that well, as described in Figure 2. Fluorescence measurements were normalized by the biomass of each culture according to dry weight to facilitate absolute comparisons across strains. Data represent the mean of two independent experiments. *A. fumigatus* exhibited the greatest extent of proteolytic activity, followed by *A. nidulans*, then *A. flavus*. IQFP probes used for the experiments in panels *B* and *C* are highlighted in red. *B*. Cleavage of selected IQFPs by three *Aspergillus* species. Values represent the mean of three strains per species. *C*. Effect of heating at 50°C for 30 min on the proteolytic activity of *A. fumigatus* H237 and *A. nidulans* A4 culture supernatants. *A. nidulans* supernatant retained >80% of activity under these conditions, whereas *A. fumigatus* supernatant retained only 30–40%. * p≤0.01 as compared to *A. fumigatus*. For panels A and B, data represent fluorescence fold change after 2 h incubation; for panel C, data represent fluorescence fold change after 1 h incubation. Error bars represent standard deviations.

In two independent experiments, we observed that protease substrate specificity patterns were consistent within species and no significant intraspecies variation was observed (Figure 7A). The lone exception was *A. fumigatus* strain CEA10, which had greater proteolytic activity than the other two *A. fumigatus* strains, although the cleavage patterns were consistent. In contrast, several important differences were observed when comparing species. Overall, proteolytic activity was generally greater in *A. fumigatus* strains, followed by *A. nidulans*, with *A. flavus* exhibiting the lowest level of proteolytic cleavage of the three species (Figure 7B). *A. terreus* proteolytic cleavage was undetectable at the dilution studied (data not shown). Differences in substrate specificities were also observed among species; cleavage preferences for the Ser/Thr-Ile/Leu-Asn/Gln and Ala/Val-Ile/Leu-Ile/Leu motifs were consistent, but interspecies differences in substrate specificities of the other two motifs were evident. While *A. fumigatus* proteases cleaved Ser/Thr-Ile/Leu-Phe/Tyr promiscuously, *A. nidulans* proteases preferred sequences containing Ile at the *Yaa* position and *A. flavus* proteases preferred sequences containing Leu at the *Yaa* position. In addition, cleavage of the Ile/Leu-Phe/Tyr-Phe/Tyr motif was detectable in culture supernatants of all three *A. flavus* isolates but almost entirely absent in *A. nidulans* culture supernatants.

## Discussion

We are investigating secreted fungal proteases as novel biomarkers for diagnosis of *AF* infection. In this regard, studies focused on individual proteases have provided limited insight, given the large number of proteases in the genome. We reasoned that a profile of overall *AF* secreted protease activity would reveal the most actively cleaved substrate motifs, addressing a significant deficit in the current literature and facilitating development of protease-targeted diagnostics. As proof-of-principle, we defined the proteolytic secretome of *AF* H237, a clinical isolate commonly used for studies of *AF* pathogenesis [43], [44]. Here, we report the identities of those substrate motifs and the characteristics of the fungal proteases that cleave them.

*Aspergillus* protease secretion is greatly affected by growth conditions, including medium composition, pH, and duration of culture [23], [24], [45], [46]. *AF* is also known to secrete a different repertoire of proteases in the lung as compared to in vitro [21], and the protease repertoire may be further altered in serum. Thus, any particular in vitro culture condition may not closely resemble the in vivo growth environment as it progresses from colonization to infection to dissemination. In selecting the growth conditions for this study, we struck a balance between two factors – mimicking the in vivo environment while simultaneously using growth conditions that were amenable to comparison with other studies of *Aspergillus* secreted proteases in the literature. A relevant biological fluid (either bronchoalveolar lavage fluid or serum) was an appropriate choice as the sole carbon/nitrogen source. We selected serum because preliminary experiments showed that *AF* grew reasonably well in human serum but very poorly in BALF (data not shown). We complemented serum with a salt solution (*Aspergillus* minimal salt solution) and buffer (pH 6.5) that facilitated direct comparison with existing literature [45], [47], [48].

### Substrate specificities of *AF* secreted serine proteases

Serine/threonine proteases are abundant in the *AF* genome and a serine protease, Alp1, is actively expressed during infection [17]. Thus, serine proteases were natural targets for our study. The virulence of mutant fungal strains lacking “Alp1-like” elastase activity is lessened considerably [17], but fungal strains in which Alp1 has been genetically deleted retain virulence [17], [26], [49], [50], [51]. Therefore, additional serine proteases may be expressed during infection [51]. Isoelectric separation of *AF* culture supernatant revealed at least six distinct fractions with serine protease activity [52], further supporting the possibility that multiple serine proteases are present during infection and may be suitable diagnostic targets.

By screening a diverse library of 3375 IQFPs we identified the substrate sequences most actively cleaved by *AF* secreted proteases under serine protease buffer conditions (pH 8.0) (Figure 2 and Table 1). Many of these substrates were not cleaved by human serum, suggesting that they may be suitable candidates for detection of *AF* protease activity in the host. Collectively, there is a significant preference for isoleucine, leucine, phenylalanine, and tyrosine in the substrates cleaved by *AF* secreted proteases. This is an important finding because the most abundant *AF* secreted serine protease, Alp1, hydrolyzes peptide bonds after both hydrophobic and cationic non-prime side residues [52]; the relative contribution of each has not been determined. The proteolytic activities we observed were inhibited by AEBSF, a serine protease inhibitor, but not by other class-specific inhibitors, suggesting that serine proteases are indeed responsible (Figure 6). In addition, Alp1 is reported to have potent collagenolytic activity, which may allow it to efficiently degrade collagen in the lung [25]. Thus, it is somewhat surprising that cleavage of proline-containing IQFPs was poor (Figure 2; Table 1). However, prolyl endopeptidase activity could occur under alternative conditions [23]. Although we do not yet know if Alp1 is responsible for the proteolytic activities reported here, the identity of the protease(s) is not essential for diagnostic assay development.

Schwienbacher and colleagues determined the major proteins secreted by *AF* strain NCTC2109 during growth in several different media [45]. In that study, the most abundant proteins secreted by *AF* during culture in AMM at 37°C were mitogillin, chitosanase Csn, chitinase ChiB1, and a ß-1,3-endoglucanase. An aspartic protease, aspergillopepsin i, was induced by growth in a more acidic medium, further underscoring the extent to which *AF* adjusts hydrolase secretion to match its environment. When Schwienbacher et al. comparatively profiled the secreted proteins of other *Aspergillus* species, they observed qualitatively different protein profiles by SDS-PAGE, consistent with the differences in protease specificity we observed among species (Figure 7A). The authors of that study also found *A. nidulans* and *A. terreus* protein secretion to be below their limit of detection, consistent with the lower levels of protease activity we observed as compared to *A. fumigatus*. We do not yet know if interspecies differences in secreted protease activity arise from alterations in specific activity or substrate specificity of particular proteases or simply from globally reduced levels of protein secretion.

Mass spectrometry-based proteomics approaches are essential tools for gaining insight into biological phenomena, including proteolysis [30], [53]. Although powerful, many of these sophisticated techniques require state-of-the-art instrumentation and laborious methodology. The method reported here allows cleavage sites to be determined in a standard MALDI-TOF mass spectrometer after a one step ZipTip® purification without the need for liquid chromatography fractionation. The three amino acid variable region allows substrate specificity to be determined rapidly with only one cleavage site per sequence. However, because this information is largely restricted to non-prime side residues (Table 3), more diverse synthetic libraries may be necessary to determine prime side specificity [54]. Emerging computational, quantitative, or targeted proteomic methods would also be useful in this regard [55], [56], [57].

Schaal and coworkers previously assayed *AF* culture supernatant against a panel of 360 protease substrates with the goal of identifying fungus-specific proteolytic activity in blood for diagnosis of *AF* infection [58]. The authors of that study identified 10 substrate sequences that were cleaved by *AF* culture supernatant but not by human serum. Several of these sequences contained aliphatic or aromatic residues, but the consensus motifs identified in our study were not explicitly present. A comprehensive comparison of our data to those reported by Schaal is difficult because the sequences assayed by Schaal were longer (eight amino acids) and thus could have multiple cleavage sites. By expanding the pool of candidate substrate sequences in an unbiased manner, our technique complements the work of Schaal and coworkers.

### Thermostability of *AF* proteases

The thermotolerance of *AF* is unique among pathogenic molds [59], and secreted hydrolases of multiple *Aspergillus* species exhibit both thermostable (resistant to irreversible inactivation at high temperature) and thermophilic (able to maintain enzymatic activity at high temperature) properties [60], [61]. These thermal properties may be useful in distinguishing their activity from that of mammalian proteases in diagnostic samples. In our study, the proteases corresponding to the most strongly cleaved substrates were thermostable up to 45°C, with activity declining sharply above that temperature (Figure 5A). This finding agrees closely with previous studies of Alp1 [25] and with studies of additional *AF* secreted serine proteases [52]. We also demonstrated that these proteases exhibit thermophilic behavior, with at least one IQFP motif demonstrating significantly greater cleavage at 45°C than at room temperature. To our knowledge, this is the first report of thermophilicity of *AF* secreted proteases. In the future, a combination of differences in thermostability and proteolytic signature could be exploited to distinguish among pathogenic *Aspergillus* species.

The observed thermostability of *A. nidulans* secreted proteases was surprising, since *A. fumigatus* has been considered uniquely thermotolerant among *Aspergillus* species [62]. However, *A. nidulans* is capable of growing at 48°C in vitro [59]. In addition, a recent report described a secreted alkaline protease from *A. nidulans* with biochemical characteristics that closely match the proteolytic activity we observed (pH optimum 8.0, thermostability up to 50°C) [63]. Taken together, these observations suggest that further investigation of the thermotolerance of *Aspergillus nidulans* is warranted.

### Relevance to pathogenesis and diagnosis

The role of secreted proteases in *AF* pathogenesis has been difficult to prove. Early studies suggested a correlation between fungal elastase activity and virulence in vivo [17], and *AF* secreted proteases have recently been implicated in fungal evasion of complement [64], [65]. However, deletion strains lacking two major *AF* secreted proteases (Alp1, Mep) do not exhibit reduced virulence in animals [26], [49], [50]. Because the *AF* genome encodes 136 proteases [22], any role of *AF* proteases in virulence may thus far have been overlooked because of redundancy in the large number of secreted proteases in the genome. The transcription factor PrtT, which drives expression of many *AF* secreted proteases in vitro, is not a virulence factor [66], [67]. However, these genetically modified strains were not completely devoid of proteolytic activity, and a role for proteases not controlled by PrtT cannot be discounted. Thus, alternative experimental approaches may provide additional insight into the role of secreted proteases in *AF* pathogenesis.

Protease activity profiling represents a new means of comparison of *Aspergillus* protease activities. Protease secretion in vitro is highly dependent on the growth conditions [23], [46], [68], but these differences have not been studied at the level of substrate specificity. This tool may also be helpful in addressing the role of *AF* proteases in virulence through correlation of proteolytic signature with pathogenicity of *AF* strains. Characterization of genetically modified strains is also possible; for example, we recently identified protease activities that are disproportionately affected in genetically modified *AF* strains that are deficient in an endoplasmic reticulum stress response pathway [47].

Substrate-based design of covalent active-site inhibitors is another field that could benefit from a more complete picture of protease activity of pathogenic organisms [30], [53]. In addition, protease activity profiling of culture supernatants, lysates, or even intact organisms may lead to the development of protease-targeted therapeutics for other organisms as well. For example, secreted aspartic proteases are confirmed virulence factors in *Candida albicans* [69]. Proteases have also been suggested as targets for therapy and diagnosis of cancer, inflammation, and numerous bacterial and viral infections [70], [71].

Our data demonstrate that IQFP protease profiling can accurately and reproducibly measure the substrate specificity and extent of cleavage of *AF* proteases within culture supernatant. To advance this technique toward development of a diagnostic assay for *AF* infection, it will be important to ascertain whether this method is sufficiently sensitive to detect *AF*-specific proteolysis in body fluids such as BALF and serum that contain abundant host proteases. Our earlier study indicated that activity of an exogenous protease is detectable in BALF at concentrations below 1 µg/mL [38]. Ultimately, protease profiling must done with biological fluids obtained from infected patients or appropriate animal models [72]. Indeed, comparative profiling of diseased and healthy fluids obtained from a guinea pig model of invasive aspergillosis has identified proteolytic motifs that are preferentially cleaved during infection (manuscript in preparation). Future studies will determine if this observation can be translated into a sensitive and specific diagnostic assay for *AF* infection.

### Summary

By using a combinatorial substrate library to comparatively profile the proteolytic activitites of human serum and the secretome of an *AF* clinical isolate, we found the two samples to have markedly distinct proteolytic signatures. We identified consensus substrate motifs of *AF* secreted proteases, revealing a striking preference for isoleucine, leucine, phenylalanine, and tyrosine. These motifs were cleaved by thermostable serine proteases, which retained activity up to 50°C. Dramatic differences in these secreted protease activities were observed between *Aspergillus* species. Therefore, an IQFP-based assay could distinguish among multiple *Aspergillus* species by identifying differences in their characteristic proteolytic signatures, and the thermostability of *AF* proteases may permit their differentiation from host enzymes. This approach will supplement the biochemist's toolkit for studying the role of proteases in disease, and the substrate motifs that we identified may be suitable targets for diagnosis of *AF*. Current efforts are focused on profiling the proteolytic signatures of serum and bronchoalveolar lavage fluid derived from experimentally infected animals to discover IQFPs that are cleaved preferentially during infection.

## Materials and Methods

### Strains and culture conditions

*A. fumigatus* H237, AF293, and CEA10 are clinical isolates [73], [74], [75]. *A. flavus* (A1120, A1168, A249), *A. nidulans* (A4, A991, A92), and *A. terreus* A1156 strains were obtained from the Fungal Genetics Stock Center at the University of Missouri. Strains were maintained on and harvested from *Aspergillus* minimal medium (AMM) agar plates. AMM is composed of a minimal salt solution (0.5% KCl, 0.5% MgSO_4_, 1.4% KH_2_PO_4_, and trace elements) supplemented with a nitrogen source (10 mM ammonium tartrate) and a carbon source (1% glucose). Culture supernatants were harvested from *Aspergillus* cultures grown as described [76] with modifications. In brief, conidia were inoculated at a concentration of 5×10^6^/mL in 50 mL of *Aspergillus* minimal salt solution, pH 6.5, supplemented with 10% heat-inactivated human serum (blood group type AB, Innovative Research, Novi, MI) as the sole carbon/nitrogen source and incubated at 37°C for 72 h at 150 rpm. The fungus was grown for 72 h because the biomass of the culture peaked at that time (data not shown). In addition, these are standard growth conditions used by our group and others to prepare fungal cultures for downstream biochemical analysis [45], [47], [48].

Cultures were centrifuged at 4°C for 5 min at 4500 rpm, decanted, and supernatants were filtered with sterilized Miracloth (EMD Chemicals). The total biomass in liquid cultures was determined by lyophilization of the fungal pellet from pre-weighed, sterile conical tubes before a final weight was recorded. In control experiments, repeated additional filtration through 200 nm syringe filters did not diminish proteolytic activity (data not shown). Supernatants were stored at 4°C and assayed within 5 days, over which time no change was observed in caseinolysis or in proteolysis of any of the peptides used in this study (data not shown). For each preparation of fungal culture supernatant, proteolytic activity was measured as described below using positive and negative control peptides to confirm consistent proteolytic activity between preparations (Figure 4B).

An initial comparison of buffer conditions for maximal proteolytic activity of culture supernatant preperations was performed using casein labeled with fluorescein isothiocyanate (FITC) as follows: 10 µL of a 100 µM solution of FITC-casein (Anaspec) in water was added to 10 µL culture supernatant (diluted 1∶10 in assay buffer) and incubated at room temperature for 5 h in the dark. After this incubation, proteins were precipitated by the addition of 50 µL 10% trichloroacetic acid, further incubated for 30 min at RT, and centrifuged at 15,000 rpm on a tabletop centrifuge for 15 min. Following centrifugation, 40 µL supernatant was diluted into 1 mL HEPES buffer (composition described below), 50 µL of each sample was added to a low-volume black microplate (Molecular Devices), and fluorescence intensity was recorded on an Analyst HT instrument (Molecular Devices) using excitation and emission filters of 485 nm and 530 nm, respectively. The following assay buffers were compared: serine protease – 50 mM HEPES, 100 mM NaCl, 10 mM CaCl_2_, pH 8.0; metalloprotease – 50 mM Tris, 100 mM NaCl, 10 mM CaCl_2_, 10 µM ZnCl_2_, pH 7.5; cysteine protease – 50 mM citrate, 100 mM NaCl, 1 mM DTT, 1 mM EDTA, pH 5.5; aspartyl protease – 50 mM citrate, 100 mM NaCl, pH 4.0.

### Protease activity profiling

Analysis of secreted protease specificities of *AF* culture supernatant was performed using a library of 3375 IQFP peptide substrates divided into 512 microtiter plate wells (Mimotopes). This library has been validated previously through substrate specificity profiling of both purified recombinant proteases and complex biological fluids [38], [77]. These probes remain optically silent in the uncleaved state, but upon cleavage they emit a fluorescent signal with intensity proportional to the extent of cleavage. Peptides in this library contain the sequence MCA-Gly-Gly-Gly-*Xaa-Yaa-Zaa*-Gly-Gly-DPA-Lys-Lys where MCA corresponds to 7-methoxycoumarin-4-acetic acid (fluorophore) and DPA corresponds to N^b^-(2,4-dinitrophenyl)-L-2,3-diaminopropionic acid (quencher) (Figure 1). Two C-terminal lysines are included to improve aqueous solubility of hydrophobic sequences. *Xaa*, *Yaa*, and *Zaa* correspond to variable residues comprised of equimolar mixtures of Ala/Val, Asp/Glu, Phe/Tyr, Ile/Leu, Lys/Arg, Asn/Gln, Ser/Thr, or Pro. Thus, each well of the library contains an equimolar mixture of up to eight individual peptides. Excluded residues either are chemically incompatible (Cys, Met), interfere with fluorescence (Trp), or are rarely found in protease cleavage sites (His).

Library wells (5 nmol total peptide per well) were dissolved in 5 µL 50% acetonitrile (ACN) in ultrapure water, further diluted in 45 µL sterile-filtered HEPES buffer (50 mM HEPES, 100 mM NaCl, 10 mM CaCl_2_, pH 8.0), and subdivided into 2 aliquots of 25 µL each. For each assay, one aliquot of this solution was transferred to low-volume black microplates. Immediately before the assay, *AF* supernatant was diluted 1∶100 in HEPES buffer. At t = 0 min, diluted fungal culture supernatant (20 µL per well) was added to the black microplates containing the IQFP library. For protease activity profiling of human serum, complement preserved human serum (Innovative Research) was thawed on ice and diluted 1∶1 in HEPES buffer. At t = 0 min, diluted serum (20 µL per well) was added to the black microplates containing the IQFP library as described above.

Time-resolved fluorescence data were obtained on an Analyst HT instrument using excitation and emission filters of 320 nm and 420 nm, respectively, with readings taken every 3 min. Endpoint fluorescence intensity fold change after 5 h at room temperature was calculated as F_final_/F_initial_. No fluorescence enhancement was observed in wells containing IQFPs only or in wells containing control culture medium (*Aspergillus* minimal salt solution plus 10% heat-inactivated human AB serum) lacking fungal supernatant (data not shown). Heatmaps were generated from these data in which each square corresponds to a single assay well of stacked 96 well microplates (Heatmap Builder, Ashley Lab, Stanford University) [78]. Unless otherwise indicated, all chemicals and reagents were obtained from Sigma or VWR.

### Protease activity assays

Proteolytic cleavage of individual IQFPs was assayed as described in the library screening procedure with modifications. IQFPs were custom-synthesized, confirmed by mass spectrometry, and provided as lyophilized powders (Mimotopes). IQFP stock solutions were prepared at 10 mM in dimethylsulfoxide (DMSO) by addition of 200–300 µL DMSO to 2–3 µmol peptide and stored at −20°C. In control experiments, the presence of organic solvents (ACN or DMSO) at the low levels used in these studies did not affect fluorescence or proteolytic cleavage (data not shown). IQFPs were diluted to 100 µM in assay buffer (HEPES buffer as described above, unless otherwise indicated) and fungal culture supernatant was diluted 1∶100 in assay buffer immediately before use. Working dilutions of IQFPs and fungal culture supernatant (25 µL each per well) were added to low-volume black microplates and time-resolved fluorescence data were recorded as described above.

For deconvolution of selected IQFP motifs, the 8 individual sequences derived from each selected well were assayed as described and fluorescence fold change after 2 h was calculated as F_final_/F_control_ where F_control_ represents the fluorescence intensity of wells containing IQFPs incubated with control culture medium lacking fungal supernatant. For this experiment, measurements were obtained in triplicate. The two IQFPs demonstrating the highest level of activity from each selected library well were selected for additional characterization. To measure the variability of *AF* secreted protease activity between fungal culture preparations, cleavage of these 8 IQFPs (2 each from 4 different motifs) was compared for 4 independent *AF* H237 supernatant preparations. Unless otherwise noted, fluorescence fold change and percent activity values for all subsequent assays were calculated at 1 h to facilitate comparison across substrates and strains because proteolytic cleavage and fluorescence of all IQFPs were in the linear range at this time point (data not shown). Percent activity relative to the appropriate control was calculated as follows, where fluorescence fold change for each sample (‘Fold’) was calculated as F_final_/F_initial_:
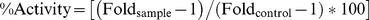
The effect of buffer pH on proteolytic cleavage of the selected substrates was determined by diluting both peptide and fungal culture supernatant in buffers of variable pH corresponding to increments of 0.5 pH units from pH 4 to 9. Buffers contained 100 mM NaCl, 10 mM CaCl_2_, and 50 mM of the buffering agent (Citrate pH 4–5.5, MES pH 6–6.5, HEPES pH 7–8, Tris pH 8.5–9). Changes in protease activity in different assay buffers are quantified as percent activity relative to the highest fluorescent signal for each individual IQFP in the assay.

Thermostability and thermophilicity of *AF* secreted proteases were determined by submerging an aliquot of culture supernatant in a heated water bath before or during proteolytic cleavage. For heating before proteolytic cleavage, undiluted culture supernatant was incubated at the temperatures and times indicated, then diluted into assay buffer and assayed as described above. For heating during the assay, undiluted culture supernatant and assay buffers were preheated at the indicated temperature for 15 min, then diluted, mixed at a 1∶1 ratio, and further heated for 1 h. At precisely 1 h, samples were removed from heat, added to microplate wells, and fluorescence readings were obtained. For all assays conducted in this manner, sample handling was staggered to ensure accurate timekeeping. Changes in protease activity at different temperatures are quantified as percent activity relative to activity at room temperature.

To determine the effect of class-specific protease inhibitors on the activity of *AF* secreted proteases, culture supernatant and IQFPs were diluted in assay buffer with the indicated concentration of inhibitor and incubated for 30 min at room temperature before starting the assay. The inhibitors were 4-(2-aminoethyl) benzenesulfonyl fluoride (AEBSF; serine proteases), ethylenediaminetetraacetic acid (EDTA; metalloproteases), E-64 (cysteine proteases), and pepstatin A (aspartic proteases). Changes in protease activity in the presence of inhibitors are quantified as percent activity relative to activity in buffer lacking inhibitor.

For comparison of proteolytic activity among *Aspergillus* strains and species, assays were performed as described with *A. fumigatus*, *A. flavus*, and *A. nidulans* supernatants diluted 1∶100 in assay buffer. In this experiment, fold change values were calculated at 2 h. To compensate for slight differences in fungal growth, fluorescence fold change values were normalized according to the dry weight of each fungal culture. The effect of heating at 50°C on proteolytic activity of *A. nidulans* A4 culture supernatant as compared to *AF* H237 culture supernatant was determined as described above.

### Determination of cleavage sites

Substrate cleavage sites were determined by identification of N-terminal and C-terminal substrate fragments by matrix-assisted laser desorption/ionization time-of-flight (MALDI-TOF) mass spectrometry (Figure 1). At the conclusion of the protease assays described above, 10 µL per well was removed, desalted, and concentrated with solid phase extraction micropipette tips (ZipTip® C_18_, Millipore, Billerica, MA) according to the manufacturer's instructions. Samples were eluted directly (1 µL each) onto a MALDI target plate and air-dried. Matrix solution (a-cyano-4-hydroxycinnamic acid in 50∶50∶0.3 water∶acetonitrile∶trifluoroacetic acid; 1 µL) was subsequently spotted on top of each sample and allowed to dry. Spectra were then acquired with an ABI 4800 MALDI-TOF/TOF mass spectrometer (Applied Biosystems, Carlsbad, CA) with an m/z range of 400–1500 Da. The instrument is routinely calibrated using the 4700 Mass Standards Kit (Applied Biosystems). Intact parent peptides were identified through an analogous procedure from control wells containing IQFPs mixed with culture media lacking fungal supernatant. Cleavage sites were assigned by comparing observed peaks with calculated expected masses corresponding to cleavage at each possible site.

### Statistical analysis

Statistical significance was assessed by analysis of variance and two-tailed Student's t-test. Differences were considered significant if they exhibited *p* values<0.05 in Student's t-test. Data analyses were performed with Microsoft Excel. All measurements were obtained in duplicate unless otherwise noted. All data are representative of at least two independent experiments performed with separate preparations of fungal culture supernatant.
